# Global trends in chronic kidney disease related cognitive impairment/dementia: a bibliometric analysis (2005–2025)

**DOI:** 10.3389/fneur.2026.1739096

**Published:** 2026-05-19

**Authors:** Ning Sun, Bo Long, Rui Huang, Xuewen Lv, Hongxiu Yang, Cheng-Qi He

**Affiliations:** 1 Rehabilitation Medicine Center and Institute of Rehabilitation Medicine, West China Hospital, Sichuan University, Chengdu, China; 2 Key Laboratory of Rehabilitation Medicine in Sichuan Province, West China Hospital, Sichuan University, Chengdu, China; 3 Department of Critical Care Medicine, The Second People's Hospital of Hunan Province (Brain Hospital of Hunan Province), Changsha, Hunan Province, China; 4 Nursing Department, The Second People's Hospital of Hunan Province (Brain Hospital of Hunan Province), Changsha, Hunan Province, China

**Keywords:** bibliometric analysis, co-citation analysis, collaboration network, keyword burst, kidney–brain axis, research trends

## Abstract

Research at the intersection of chronic kidney disease (CKD) and cognitive impairment/dementia has received increasing attention in recent years. This study conducted a bibliometric analysis of 7,971 English-language articles and reviews published between 2005 and 2025 and retrieved from Web of Science and Scopus. VOSviewer and CiteSpace were used for bibliometric visualization and network analysis. Publication trends, contributing countries/regions, institutions, authors, core journals, co-citation patterns, and keyword evolution were systematically examined. The included publications involved 12,719 authors from 150 countries to 13,280 institutions. Hooper SR and Kurella Tamura were among the most influential contributors in terms of productivity and impact. PLOS ONE had the highest number of publications, whereas The Lancet was the most frequently co-cited journal. Since 2014, 18 highly influential references have shown sustained citation bursts, mainly related to the kidney–brain axis and clinical outcomes. Keyword trends shifted from early themes such as dialysis and survival to cognitive health and quality of life, with recent emphasis on machine learning, electronic health records, and patient-centered care. Overall, the field has shown continuous growth, with North America and Europe occupying central positions in the collaboration network and with emerging trends indicating increasing attention to data-driven methods and patient-centered approaches.

## 1 Introduction

Chronic kidney disease (CKD) is defined by abnormalities of kidney structure or function persisting for more than 3 months (1). It is a major global public health problem, affecting more than 694.7 million people worldwide, with an overall prevalence exceeding 9.1% and approximately 1.2 million deaths annually directly attributable to the disease (2). Over recent decades, the mortality and overall disease burden of CKD have continued to increase (3, 4), and CKD is projected to become the fifth leading cause of death worldwide by 2040 (5). At the same time, cognitive impairment and dementia are becoming increasingly prevalent in aging populations and have emerged as major public health challenges (6). An estimated 57 million people were living with dementia in 2019, and this number is projected to approach 150 million by 2050 (7). These two conditions substantially overlap in the general population. Older adults with CKD have a markedly higher prevalence of cognitive impairment (8, 9), and the clinical significance of the overlap between kidney dysfunction and cognitive decline is receiving increasing attention (10).

Previous studies have shown that individuals with CKD, particularly those receiving dialysis, are at increased risk of cognitive impairment (11). In patients with an estimated glomerular filtration rate (eGFR) below 60 ml/min/1.73 m^2^, each 10 ml/min/1.73 m^2^ decrease in eGFR has been associated with an 11% higher prevalence of cognitive impairment. Severe cognitive impairment is reported to be approximately three times more common in patients undergoing hemodialysis than in age-matched individuals without dialysis (12). These findings underscore the importance of investigating the relationship between CKD and cognitive impairment/dementia. However, this relationship has long been underrecognized and has been described as part of the “neglected kidney–brain axis” (13). Historically, nephrology research has focused more on cardiovascular complications than on the neurocognitive consequences of CKD (14). Although a growing number of clinical and epidemiological studies have documented the prevalence and potential mechanisms of cognitive decline in CKD (15, 16), the literature in this cross-disciplinary field remains fragmented, highlighting the need for a more systematic bibliometric assessment.

In recent years, bibliometric methods have been increasingly used to investigate research trends across multidisciplinary medical fields (17). Through quantitative analysis of publication output, citation patterns, and collaboration networks, bibliometric analysis can reveal the developmental trajectory, research hotspots, and academic cooperation patterns of a field (18). However, existing bibliometric work on CKD and cognitive impairment/dementia has been limited to single databases or narrow subsets, such as the top 100 most-cited papers, and has not systematically identified emerging fronts through integrated keyword burst detection and co-citation clustering (19). As a result, the knowledge structure, thematic evolution, and collaboration landscape of this interdisciplinary area have not yet been comprehensively characterized. In contrast, bibliometric approaches have been successfully applied in other cross-disciplinary medical fields, such as Alzheimer's disease comorbidities, to map global research profiles and highlight major themes and future directions (20). Therefore, a comprehensive bibliometric analysis is needed to better understand the research landscape at the intersection of CKD and cognitive impairment/dementia.

Using integrated records from web of science core collection (WOSCC, https://www.webofscience.com/) and Scopus (https://www.scopus.com/), this study aimed to: (i) chart temporal trends and developmental phases in the CKD–cognition literature; (ii) map the research landscape and collaboration patterns, including leading authors, institutions, countries, co-authorship networks, international collaborations, core journals, and co-citation structures; and (iii) identify research hotspots and emerging fronts through keyword co-occurrence, clustering, and burst detection.

## 2 Materials and methods

### 2.1 Data source and search strategy

On June 25, 2025, we searched the WOSCC and Scopus for English-language articles and reviews published between January 1, 2005 and March 31, 2025. The search strategy consisted of two concept groups combined with the AND operator: one related to CKD and the other related to cognitive impairment/dementia. In Web of Science, the search was performed in the Topic field (TS), whereas in Scopus, the equivalent search was conducted in the TITLE-ABS-KEY field using database-specific syntax. WOSCC and Scopus were selected as the primary data sources because both provide structured bibliometric metadata, including cited references, author affiliations, and keywords, which are essential for co-citation analysis, collaboration network mapping, and keyword burst detection. The potential impact of this database selection on literature coverage is addressed in the limitations section. The complete search strings for both databases, together with all applied limits and filters, are provided in Supplementary Table S1.

The full Web of Science search string was as follows: TS = ((“Chronic Kidney Insufficien^*^” OR “Chronic Kidney Disease” OR “CKD” OR “Chronic Renal Disease” OR “Chronic Renal Insufficien^*^” OR “Kidney Failure” OR “Renal Failure” OR “Diabetic Kidney Disease” OR “Diabetic Chronic Kidney Disease” OR “Diabetic Nephropath^*^” OR “Diabetic Renal Disease”) AND (“cognit^*^” OR “cognitive impairment^*^” OR “cognitive decline” OR “cognitive dysfunction^*^” OR “cognitive deficit^*^” OR “neurocognit^*^” OR “executive function^*^” OR “dementia” OR “Alzheimer” OR “mild cognitive impairment” OR “MCI”)).

**Figure 1:**
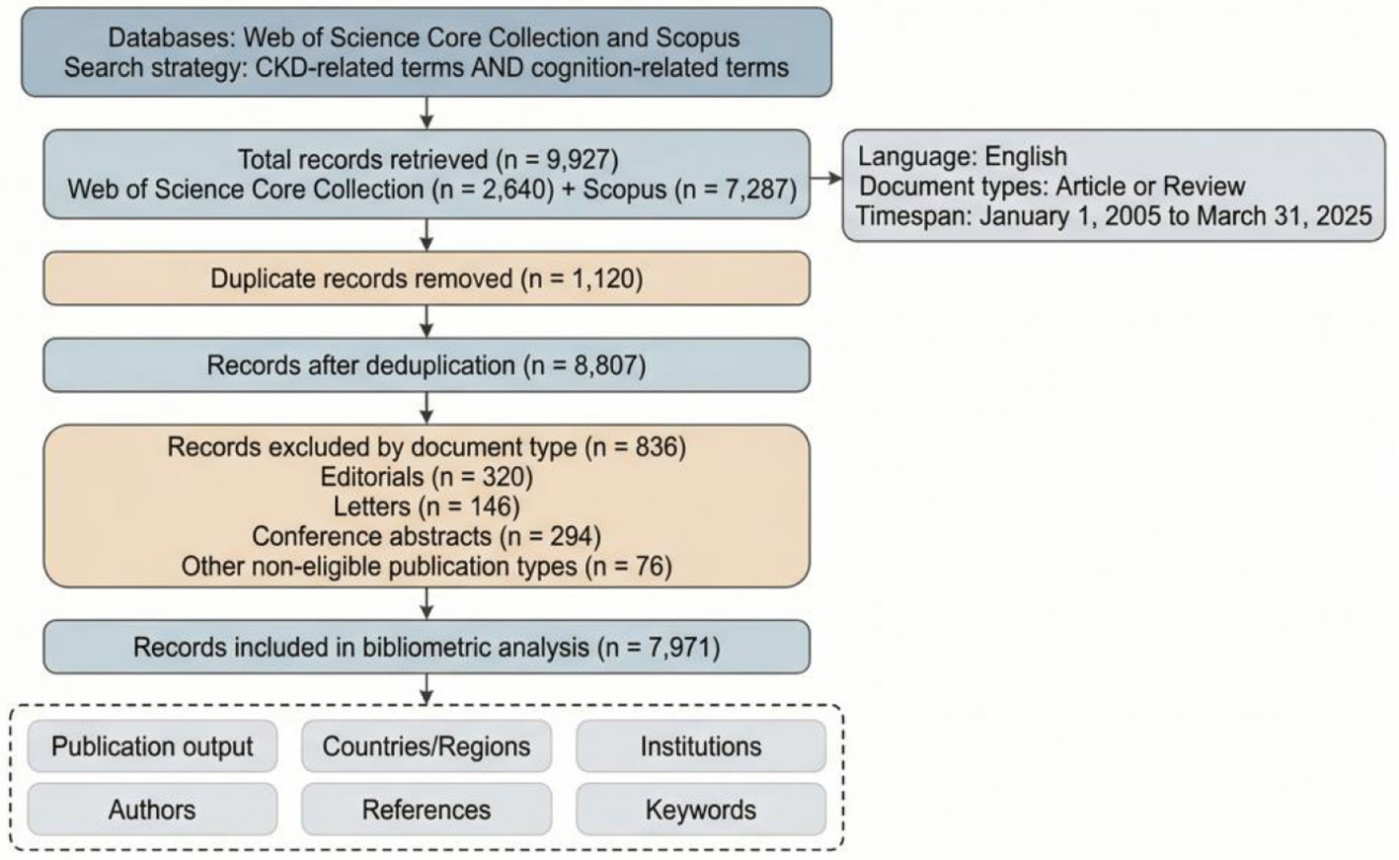
Flowchart for identifying and selecting publications.

The initial search yielded 2,640 records from Web of Science and 7,287 records from Scopus, for a total of 9,927 records. Records from the two databases were merged in EndNote. A total of 1,120 duplicate records were removed automatically and then manually verified based on DOI, title, and author/year information, leaving 8,807 records for screening. Records were then screened by document type, and 836 records were excluded, including editorials (*n* = 320), letters (*n* = 146), conference abstracts (*n* = 294), and other non-eligible publication types (*n* = 76). A total of 7,971 records were ultimately included in the bibliometric analysis (Figure 1).

### 2.2 . Analyzing and visualizing data

Three main tools were used in this study: Microsoft Excel 2019, CiteSpace (version 6.4.R1), and VOSviewer (version 1.6.20). Microsoft Excel 2019 was used as an auxiliary tool to summarize publication data, rank bibliometric indicators, and generate charts showing annual publication trends and cumulative growth. VOSviewer and CiteSpace were used for bibliometric network construction and visualization (21–23).

CiteSpace (version 6.4.R1) was primarily used for co-citation and keyword analyses. The parameters were set as follows: time span, 2005–2025; time slice, 1 year; node types, cited documents and keywords; link strength, cosine; and selection criterion, top 50 items per slice. Network pruning was performed using the Pathfinder algorithm. Clusters were generated using the Louvain algorithm, and cluster labels were extracted using the log-likelihood ratio (LLR) method. The visual outputs included static cluster maps and timeline views. In the visualized networks, node colors represent publication year, ranging from blue for earlier publications to red for more recent publications. VOSviewer (version 1.6.20) was used to construct and visualize collaboration networks for countries/regions, institutions, and authors, as well as keyword co-occurrence maps. Co-authorship analysis was performed for countries/regions, institutions, and authors, and co-occurrence analysis was performed for keywords. Full counting was applied in all VOSviewer analyses, and association strength was used as the normalization method. The minimum number of documents for countries/regions, institutions, and authors was set to 5, and the minimum number of occurrences for keywords was set to 5. Only items meeting these thresholds were included in the final visualization.

## 3 Results

### 3.1 Analysis of publications

**Figure 2:**
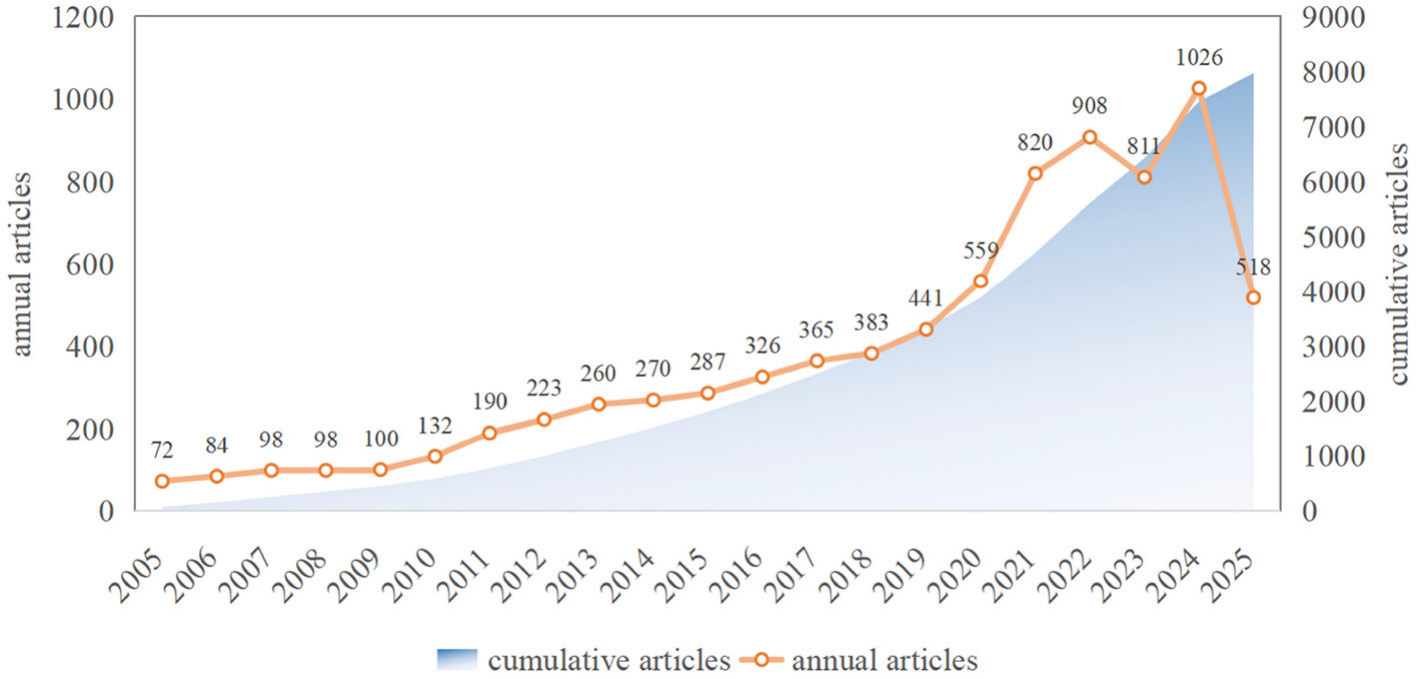
Trends in annual output and cumulative volume for CKD and cognition research.

A total of 7,971 papers on CKD and cognitive impairment/dementia were included in the study. Figure 2 displays the annual publication trend. The annual publication volume exhibited a general rising trend from 2005 to 2025, with growth rates beginning to significantly accelerate in 2020 and 2022. The publishing volume increased significantly in 2024, peaking at 1,026 papers annually. The overall trend suggests three broad phases: slow growth (2005–2013, annual output below 300), gradual acceleration (2014–2019), and rapid expansion since 2020.

### 3.2 Analysis of countries/regions

**Figure 3:**
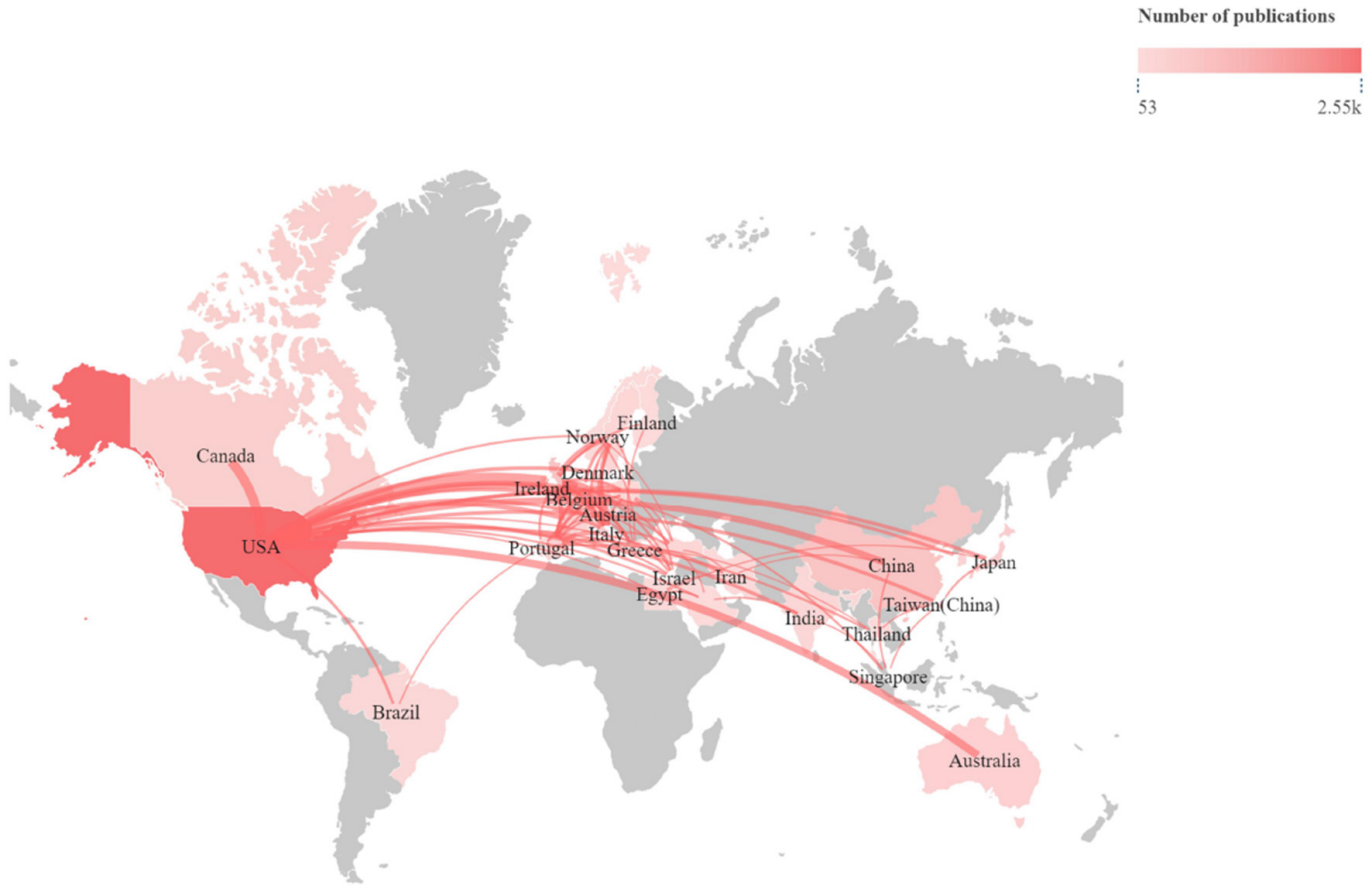
Global map of national cooperation.

**Figure 4:**
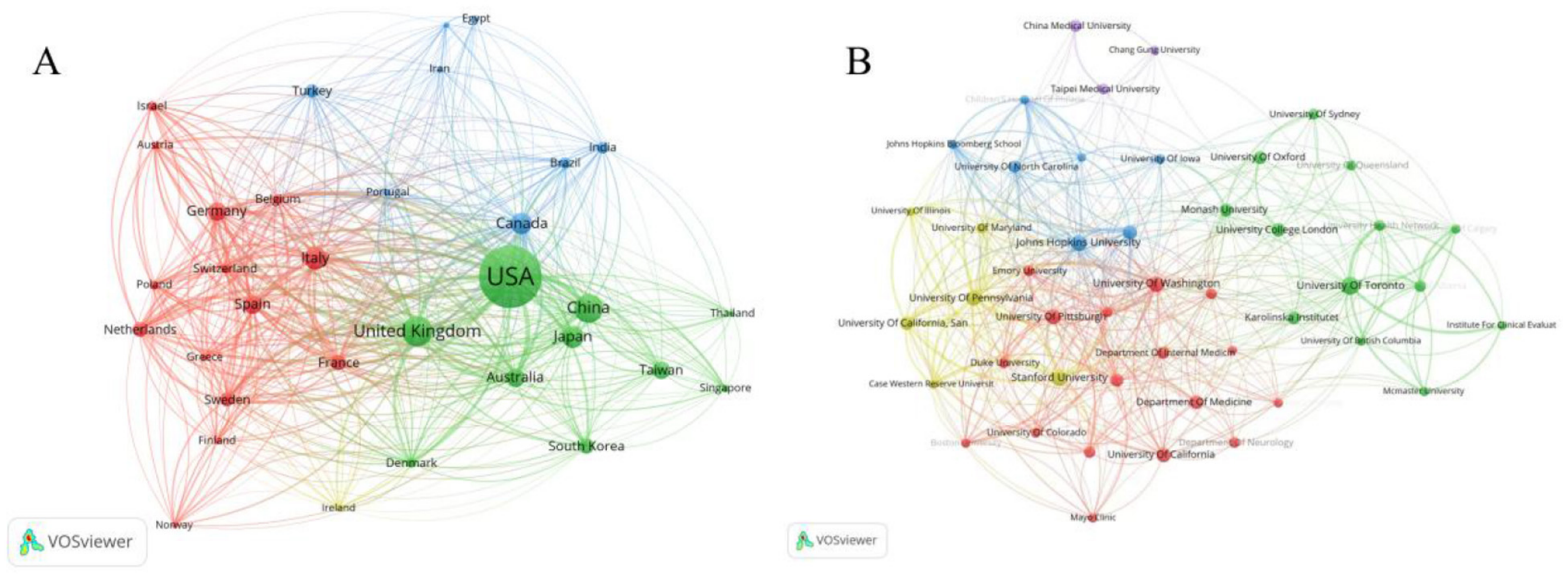
Collaboration networks of countries/regions and institutions. **(A)** Cooperation network of countries/regions. **(B)** Collaboration network of institutions.

A total of 150 countries participated in research in this field. Table 1 lists the top 10 nations by publishing volume, with the USA (*n* = 2,621), China (*n* = 1,147), and the UK (*n* = 729) coming in first, second, and third, respectively. Among the top 10 countries, China (0.09), Australia (0.08), and the USA (0.07) exhibited the highest betweenness centrality values, suggesting that these countries may occupy relatively important bridging positions within the collaboration network. The United States showed extensive international collaboration, particularly with countries in Europe and Australia, as shown in Figure 3. The nations that publish papers in this area have grouped into three clusters (Figure 4A). The red cluster is mainly made up of European nations like Germany, Italy, and Spain; the blue cluster is a collaborative network headed by nations like Canada, India, and Brazil; and the green cluster is a closely knit collaborative publishing network centered around the United States, China, and the United Kingdom. China ranks second in publication volume, but its international co-authorship network remains relatively limited compared to those of the USA and European countries.

**Table 1:** Top 10 countries/regions by number of publications.

Rank	Countries	Counts	Betweenness centrality
1	United States	2,621	0.07
2	China	1,147	0.09
3	United Kingdom	729	0.04
4	Italy	513	0.03
5	Japan	482	0
6	Canada	468	0.03
7	Australia	371	0.08
8	Spain	357	0.01
9	Germany	357	0.03
10	France	287	0.01

### 3.3 Analysis of institutions

In total, 13,280 institutions worldwide contributed to the literature in the field of CKD and cognitive impairment/dementia. Table 2 lists the top 10 institutions, with the top three being the University of Toronto (*n* = 126), Johns Hopkins University (*n* = 103), and the University of Washington (*n* = 103). Notably, eight of the top 10 institutions were from the United States, indicating the prominent role of US institutions in this research field. Five groups were formed by the publishing institutions (Figure 4B). The purple cluster is centered around “China Medical University” and “Chang Gung University” which are primarily concentrated in China; the red cluster is made up of a group of US institutions centered around the University of Washington; the green cluster is primarily made up of a group of Canadian institutions centered around the University of Toronto; and the yellow and blue clusters, respectively, form collaborative networks led by the “University of California” and “Johns Hopkins University”, which are primarily concentrated in the United States. These five clusters are largely organized along geographic lines, with four clusters concentrated in North American institutions and one centered on East Asian institutions, suggesting that institutional collaboration in this field remains predominantly regional.

**Table 2:** Top 10 institutions by number of publications.

Rank	Institutions	Counts	Country
1	University of Toronto	126	Canada
2	Johns Hopkins University	103	United States
3	University of Washington	103	United States
4	Stanford University	96	United States
5	University of Pennsylvania	92	United States
6	University of Pittsburgh	91	United States
7	University of California	85	United States
8	University of Michigan	84	United States
9	University of California, San Francisco	75	United States
10	University College London	73	United Kingdom

### 3.4 Analysis of authors and co-authors

A total of 12,719 authors contributed to the literature on CKD and cognitive impairment/dementia. As shown in Table 3, Hooper SR ranked first with 34 publications, followed by Kurella Tamura M and Furth SL with 25 and 23 publications, respectively. Among co-cited authors, Levey AS, Kurella Tamura M, and Murray AM ranked in the top three, suggesting that their work has had substantial influence in this field.

**Table 3:** Top 10 productive authors and co-cited authors in this field.

Rank	Authors	Counts	Rank	co-cited authors	Counts
1	Hooper SR	34	1	Levey AS	544
2	Kurella Tamura M	25	2	Kurella Tamura M	509
3	Furth SL	23	3	Murray AM	470
4	Corsonello A	23	4	Charlson ME	424
5	Murray AM	22	5	Folstein MF	357
6	Formiga F	19	6	Drew DA	294
7	Lattanzio F	18	7	Yaffe K	280
8	Warady BA	18	8	Fried LP	251
9	Lip GYH	18	9	Griva K	234
10	Yaffe K	17	10	Bugnicourt JM	223

### 3.5 Analysis of journals

A total of 2,222 scholarly journals were included in this domain. The top 10 journals in terms of both total citations and publication volume are shown in Table 4. According to the Journal Citation Reports (JCR), the majority of journals are categorized in the first and second quartiles. *PLOS ONE* has the most publications (*n* = 145), followed by *BMC Nephrology* and *BMJ Open*. *The Lancet* has the most citations and the highest impact factor among the top ten most referenced journals. However, we also observe that *PLOS ONE* is in the top three for both the quantity of articles published and the quantity of citations.

**Table 4:** Top 10 journals and co-cited journals.

Rank	Journal	Total counts	IF	JCR	Rank	Co-cited journal	Total cites	IF	JCR
1	PLOS ONE	145	2.9	Q2	1	The Lancet	2,077	98.4	Q1
2	BMC Nephrology	109	2.2	Q2	2	PLOS ONE	1,873	2.9	Q2
3	BMJ Open	108	2.4	Q2	3	American Journal of Kidney Diseases	1,857	9.4	Q1
4	Clinical Journal of the American Society of Nephrology	104	8.5	Q1	4	Journal of the American Geriatrics Society	1,640	4.3	Q1
5	Journal of Clinical Medicine	94	3	Q1	5	Kidney International	1,613	14.8	Q1
6	American Journal of Kidney Diseases	93	9.4	Q1	6	Neurology	1,534	8.4	Q1
7	Journal of the American Geriatrics Society	83	4.3	Q1	7	Journal of the American Society of Nephrology	1,489	10.3	Q1
8	Nephrology Dialysis Transplantation	75	4.8	Q1	8	Annals of Internal Medicine	1,329	19.6	Q1
9	Frontiers in Medicine	44	3.1	Q1	9	Journal of the American Medical Association	1,305	55	Q1
10	Journal of the American Medical Directors Association	44	4.2	Q2	10	New England Journal of Medicine	1,300	96.3	Q1

### 3.6 Analysis of references

**Figure 5:**
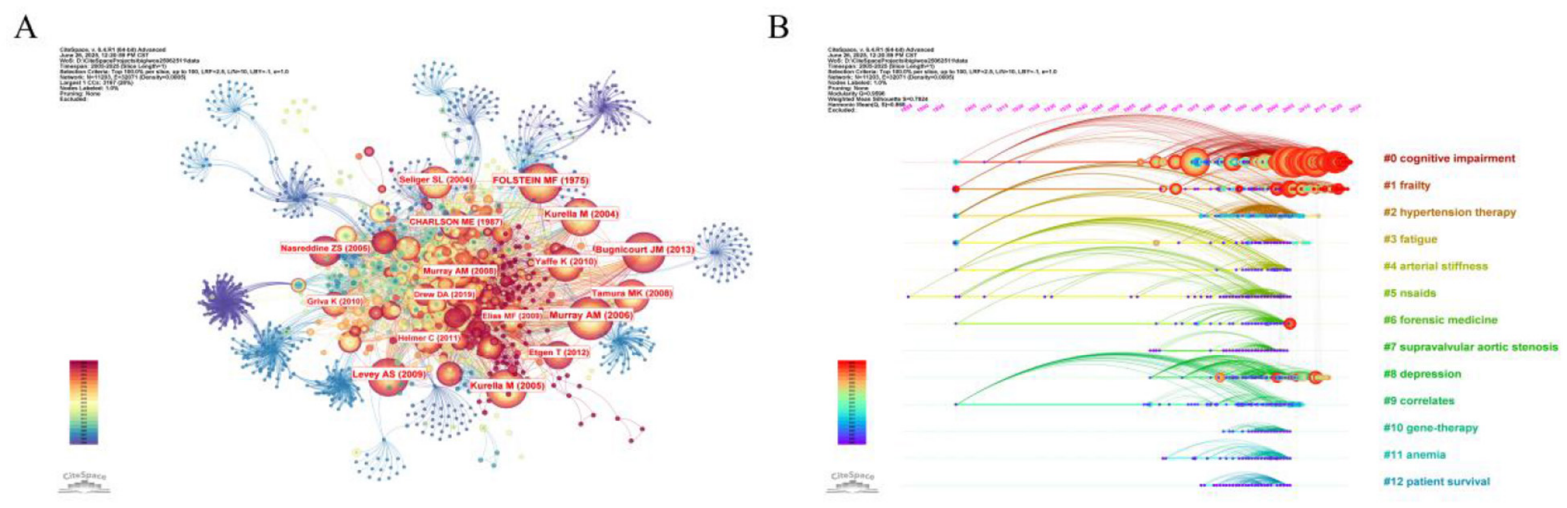
The analysis of references. **(A)** Co-citation network of cited references. **(B)** Clustering timeline of cited references.

Table 5 lists the 10 most referenced references, which include seven articles and three reviews. Up to 73.4% of hemodialysis patients with moderate to severe cognitive impairment went undiagnosed, according to a 2006 study by Murray et al. in Neurology, which was cited 215 times and ranked first. This study highlights the importance of routine cognitive screening in the dialysis population. Second place goes to Kurella et al.‘s 2004 study, which was co-cited 190 times. It was the first to show a graded correlation between cognitive impairment and declining kidney function, supporting the need for early CKD care. Third, Folstein et al. (1975) presented the MMSE scale, which provided a standardized assessment framework for future cognitive function research. Figures 5A, B show the co-citation network and clustering timeline of cited references. The CiteSpace timeline clustering diagram revealed 13 co-citation clusters reflecting the temporal evolution of the field's knowledge base. The earliest active clusters, #12 (patient survival) and #11 (anemia), were most prominent before 2010 and correspond to the initial focus on dialysis outcomes. Clusters #4 (arterial stiffness) and #5 (NSAIDs) emerged during 2010–2015, reflecting growing attention to vascular pathology and drug safety. In the most recent period, clusters #0 (cognitive impairment) and #1 (frailty) have become the dominant active themes, while cluster #8 (depression) indicates increasing recognition of the psychiatric dimensions of CKD-related cognitive decline.

**Table 5:** The top 10 cited references in the field.

Rank	Cited reference	Counts
1	Murray AM, 2006, NEUROLOGY, V67, P216, DOI 10.1212/01.wnl.0000225182.15532.40	215
2	Kurella M, 2004, J AM GERIATR SOC, V52, P1863, DOI 10.1111/j.1532-5415.2004.52508.x	190
3	Folstein MF, 1975, J PSYCHIAT RES, V12, P189, DOI 10.1016/0022-3956(75)90026-6	187
4	Bugnicourt JM, 2013, J AM SOC NEPHROL, V24, P353, DOI 10.1681/ASN.2012050536	185
5	Kurella M, 2005, J AM SOC NEPHROL, V16, P2127, DOI 10.1681/ASN.2005010005	185
6	Yaffe K, 2010, J AM GERIATR SOC, V58, P338, DOI 10.1111/j.1532-5415.2009.02670.x	138
7	Seliger SL, 2004, J AM SOC NEPHROL, V15, P1904, DOI 10.1097/01.asn.0000131529.60019.fa	132
8	Etgen T, 2012, AM J NEPHROL, V35, P474, DOI 10.1159/000338135	109
9	Murray AM, 2008, ADV CHRONIC KIDNEY D, V15, P123, DOI 10.1053/j.ackd.2008.01.010	108
10	Drew DA, 2019, AM J KIDNEY DIS, V74, P782, DOI 10.1053/j.ajkd.2019.05.017	85

**Figure 6:**
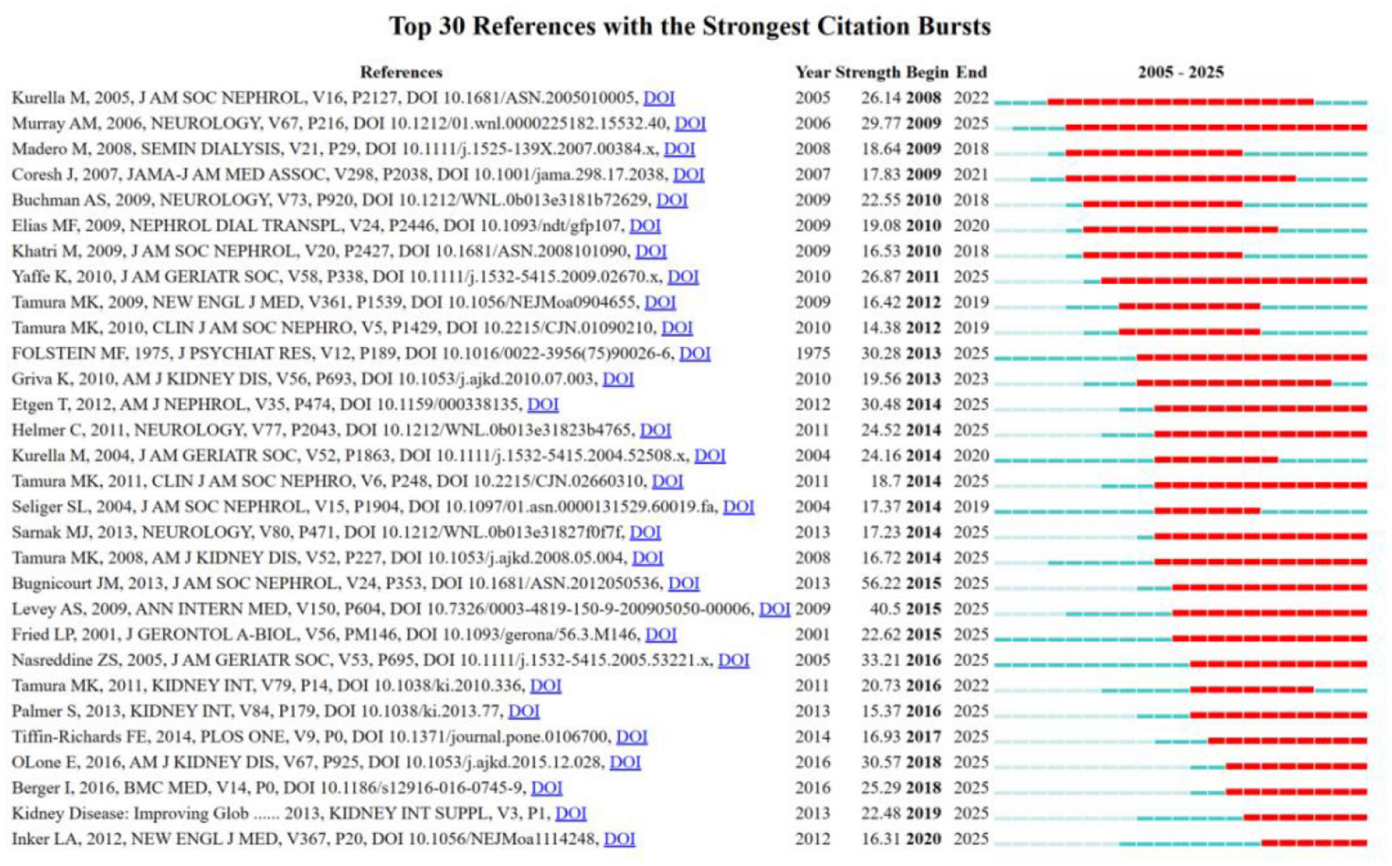
Top 30 references with the strongest citation bursts.

According to the top 30 references with the strongest citation bursts (Figure 6), 18 references showed sustained citation bursts after 2014. With an outbreak intensity of 56.22 (2015–2025), Bugnicourt JM (2013) comes in first place among these, proposing the “kidney–brain axis” framework linking CKD to cognitive impairment. Levey AS (2009, intensity 40.5, 2015–2025) comes in second, emphasizing the contribution of updated eGFR assessment criteria to the advancement of cognitive research. Among the other high-impact highlights are Folstein MF (1975, intensity 30.28), Etgen T (2012, intensity 30.48), Nasreddine ZS (2005, intensity 33.21), and O'Lone F (2016, intensity 30.57). The fact that several papers from research teams, including Kurella M, Murray AM, and Tamura MK, have been on the outbreak list several times in various years, the longest outbreak window being more than 10 years, highlights their fundamental and long-lasting impact on the field of CKD cognition.

### 3.7 Analysis of keywords

**Figure 7:**
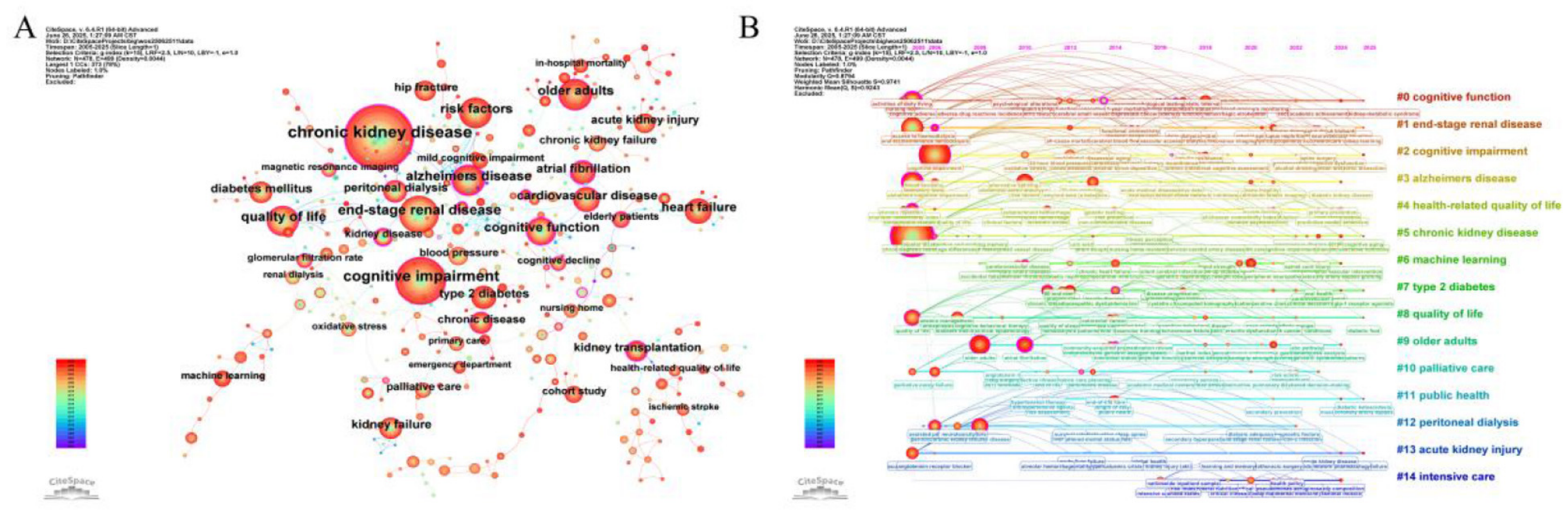
Analysis of keywords. **(A)** Co-occurrence network of keywords **(B)** Clustering timeline of keywords.

Research hotspots and innovative advancements in the field are reflected in keywords, which serve as a fundamental synopsis and focal point of a literature study's research material (24). Figure 7A is the co-occurrence network map of keywords and the top 20 keywords are listed in Table 6. The top three terms were risk factors, quality of life, and end-stage renal disease after search terms were eliminated. An important node is indicated by a centrality value higher than 0.1. 11 of these keywords, such as Alzheimer's disease, cognitive function, and CKD, had centrality values higher than 0.1. Figure 7B displays the keyword clustering timeline diagram. CiteSpace identified 15 keyword clusters; their fundamental structure comprised: #0 cognitive function, #1 end-stage renal disease, #2 cognitive impairment, and #3 Alzheimer's disease. These clusters broadly reflect a temporal thematic shift: earlier clusters (#1 end-stage renal disease, #7 type 2 diabetes) focused on dialysis-related outcomes, mid-period clusters (#4 health-related quality of life, #2 cognitive impairment) marked the emergence of cognitive outcomes as a distinct theme, and recent clusters (#6 machine learning, #10 palliative care, #11 public health) indicate growing methodological diversification and a broader public health framing. The research emphasis has moved from early worries about dialysis and survival rates to quality of life and cognitive health, while integrating AI prediction and interdisciplinary cooperation.

**Table 6:** The top 20 keywords.

Rank	Keywords	Betweenness centrality	Rank	Keywords	Betweenness centrality
1	chronic kidney disease	0.26	11	type 2 diabetes	0.03
2	cognitive impairment	0.12	12	kidney failure	0.08
3	end-stage renal disease	0.08	13	diabetes mellitus	0.03
4	Alzheimer's disease	0.33	14	atrial fibrillation	0.22
5	quality of life	0.18	15	acute kidney injury	0.09
6	risk factors	0.03	16	peritoneal dialysis	0.19
7	older adults	0.14	17	kidney transplantation	0.22
8	cognitive function	0.32	18	hip fracture	0.00
9	heart failure	0.04	19	chronic disease	0.10
10	cardiovascular disease	0.16	20	palliative care	0.08

**Figure 8:**
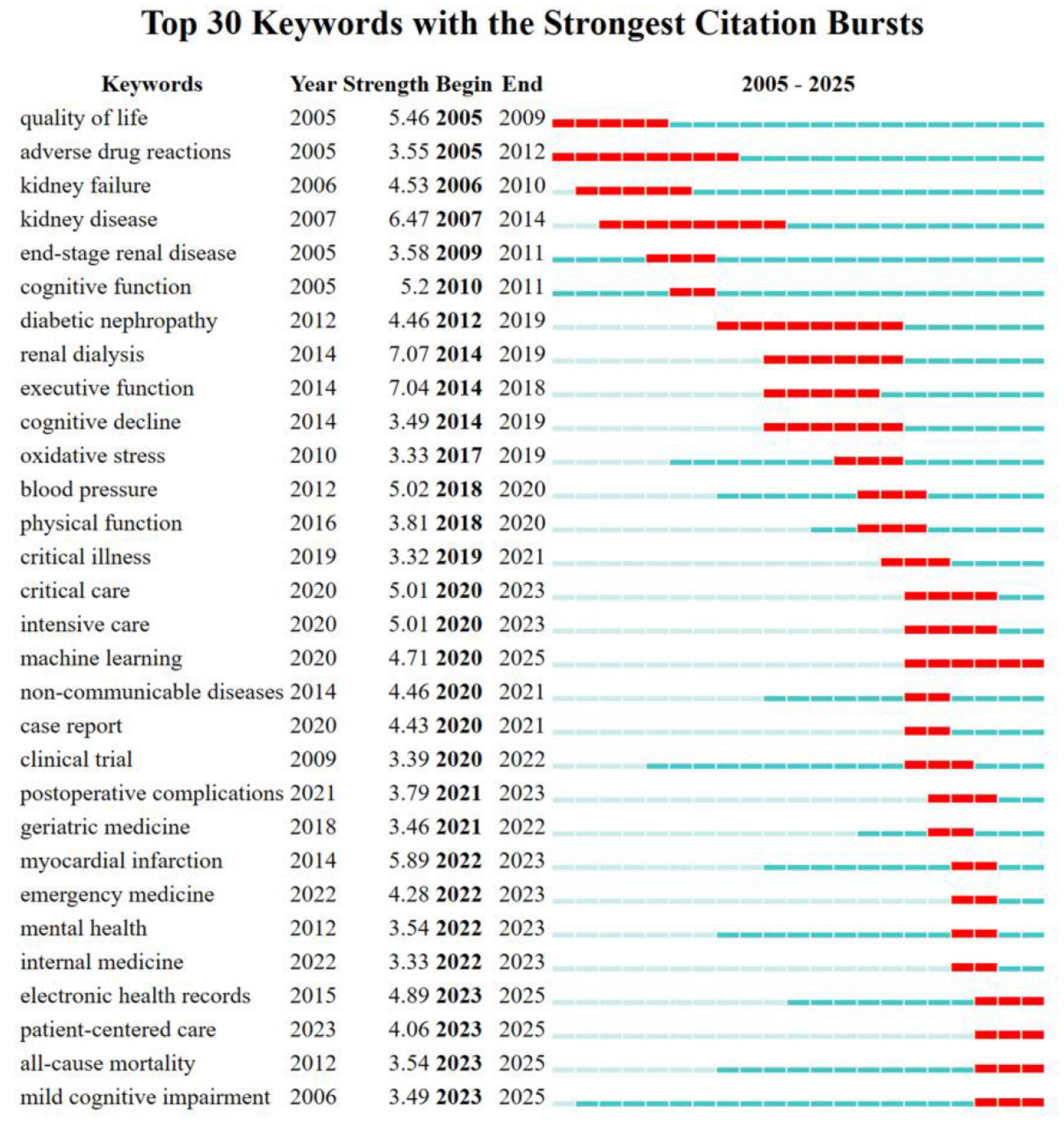
Top 30 keywords with the strongest citation bursts.

The shifting patterns in this field's research hotspots are depicted by the keyword bursts analysis (Figure 8). Research hotspots shifted from survival and treatment safety (2005–2014) through disease assessment and vascular risk factors (2014–2020) to data-driven methodologies in the most recent period, with “machine learning” (intensity 4.71, 2020–2025) and “electronic health records” (intensity 4.89, 2015–2025) emerging as prominent terms. Themes such as “patient-centered care” (intensity 4.06, 2023–2025) and “mild cognitive impairment” (intensity 3.49, 2023–2025) further indicate a broadening of research focus beyond disease-specific endpoints.

## 4 Discussion

### 4.1 Analysis of the overall trend

The annual publication volume of cross-disciplinary research on CKDs and cognitive impairment/dementia has steadily increased from 2005 to 2025, with a significant acceleration in growth after 2020, reaching a peak in 2024. This surge may be related to several contextual factors, including the global increase in aging populations, rising CKD prevalence, and the growing recognition of kidney-brain interactions in clinical research (25, 26). Concurrently, increased research funding and the formation of interdisciplinary teams have provided the necessary human and technical resources for such studies. The temporal overlap between the post-2020 publication surge and the growing availability of machine learning tools and electronic health record databases suggests that methodological advances may also have contributed to the expansion of this field, although bibliometric data alone cannot confirm this association (27–29). Taken together, these trends may reflect a gradual shift in the field from epidemiological description toward mechanism-oriented and risk-prediction research.

The United States leads the collaborative network with over 2,600 papers, forming a high-density interconnected network with high-income countries such as Europe and Australia, making it the core hub of the international collaborative network (30). China ranks second with approximately 1,147 papers, reflecting increased domestic investment and a growing burden of CKD (31), but cross-border collaboration remains insufficient. Countries such as the United Kingdom and Nordic nations form a tight cluster around the United States. Eight of the top ten high-output institutions are located in the United States, with the University of Toronto reflecting Canada's active role in geriatric nephrology (32, 33). Among productive authors, Hooper SR and Kurella Tamura M were particularly prominent, indicating the sustained contribution of several key research groups (34, 35). In addition, the prominence of authors such as Levey AS and Yaffe K in the collaboration and co-citation networks suggests that interdisciplinary collaboration has played an important role in shaping this field (36, 37). Both CKD and dementia are global public health issues (38), with large CKD patient populations in regions such as South Asia, Africa, and Latin America, yet these areas are underrepresented in the literature. Future efforts should focus on building a more globally interconnected research community.

### 4.2 Theoretical foundation of the study

The knowledge base of this field is reflected in a series of highly co-cited landmark studies that collectively illustrate the evolution of research on CKD and cognitive impairment/dementia. Early highly cited studies drew attention to the substantial burden of cognitive impairment in patients with CKD, particularly among those receiving dialysis. For example, Murray et al. reported a high prevalence of undiagnosed cognitive impairment in a large dialysis cohort, highlighting the clinical importance of routine cognitive assessment in this population (39). In addition, the inclusion of Folstein et al. among the top co-cited references indicates the central role of standardized cognitive assessment tools in this field. Together, these highly cited references suggest that early research was largely centered on disease burden recognition and cognitive screening.

As the field developed, co-citation patterns indicate a gradual shift toward more conceptually and mechanistically oriented research. Bugnicourt et al. (40), which showed the strongest citation burst, proposed the “kidney–brain axis” framework and has served as an important conceptual reference for subsequent studies. The sustained burst intensity of this reference suggests that it has continued to shape the research agenda in this area. In parallel, the co-citation clusters indicate increasing attention to potential mechanisms linking CKD to cognitive decline, suggesting that mechanistic investigation has become a more active research stream over time.

Longitudinal evidence has also become more prominent in the highly co-cited literature. For example, Zijlstra et al. reported that mild to moderate CKD was associated with accelerated decline in executive function even after adjustment for conventional cardiovascular risk factors (41). Its presence within the co-citation network suggests that the independence of CKD-related cognitive effects from traditional vascular pathways has become an important topic of discussion in the field. Overall, the co-citation patterns suggest that research at the intersection of CKD and cognitive impairment/dementia has progressively evolved from epidemiological observation and cognitive assessment toward more mechanism-oriented investigation.

### 4.3 Emerging trends and future directions

This study not only documents past trends in CKD and cognitive impairment/dementia research but also reveals emerging research areas that may influence the field in the future. Keyword co-occurrence analysis and sudden trend analysis indicate that research priorities have shifted over time: early studies primarily focused on problem definition, with keywords such as “end-stage renal disease” and “cognitive function” as core themes. In recent years, however, new concepts and cutting-edge research methods have emerged. Compared to previous studies, significant differences are evident in the expansion of research methods and areas of focus, with the rise of machine learning and big data analysis in this field (27). As indicated by the keyword burst analysis, “machine learning” and “electronic health records” are among the terms with the strongest burst intensities in the most recent period. Recent studies have applied machine learning algorithms to improve risk prediction in CKD populations (28) and leveraged electronic health record data for earlier detection of cognitive decline (29). For example, new studies have applied machine learning algorithms to improve risk prediction or identify kidney disease in CKD patients, demonstrating significant improvements in data processing and analysis capabilities compared to traditional methods (42). The integration of health record data with artificial intelligence has also facilitated early detection of cognitive impairment, which is critical for timely intervention (43). These trends suggest that predictive modeling and digital tools may become increasingly important in future research on CKD-related cognitive impairment. They also indicate growing interest in more individualized approaches to risk stratification and cognitive monitoring in CKD populations.

From a research perspective, traditional studies have primarily focused on the disease itself, while recent years have emphasized patient-centered and holistic care for patients at the intersection of CKD and cognitive impairment (44). Keywords such as “quality of life” (45), “patient-centered care” (46), and “palliative care” (47) have increasingly gained attention, reflecting a shift in research focus from disease-centered to patient-centered approaches. The emergence of “palliative care” indicates that the medical community has begun to incorporate cognitive impairment in advanced CKD and dialysis patients into comprehensive care planning (48). For example, how cognitive decline affects decision-making ability, the continuity of dialysis, or eligibility for transplantation. Meanwhile, “public health” (49) as a new keyword highlights the recognition that CKD-related cognitive impairment is not only an individual clinical issue but also a public health challenge. Taken together, these trends may indicate growing interest in cognitive screening, caregiver support, and more integrated care pathways in CKD populations. They may also suggest that future research will increasingly examine targeted cognitive assessment strategies and multidisciplinary management approaches, including collaboration across nephrology, geriatrics, and psychiatry (50, 51).

In recent years, several new concepts and treatment directions that have not been adequately addressed in previous literature have emerged. Beyond the trends directly identified through keyword burst analysis, the co-cited literature suggests growing interest in novel intervention strategies, such as uremic toxin-targeted therapies and anti-inflammatory approaches (52, 53). Although these specific topics have not yet reached the threshold of keyword burst detection, their presence in the co-citation network may signal early-stage research fronts. Another frontier is addressing CKD-related accelerated brain aging and neurodegeneration. Researchers have identified similarities between CKD-related cognitive impairment and Alzheimer's disease pathology, sparking interest in whether CKD may induce Alzheimer's-like changes or interact with amyloid metabolism (54). Consequently, there are calls to test the neuroprotective effects of interventions such as exercise and enhanced dialysis strategies. Recent individual publications have also proposed exploratory concepts such as senolytic therapies and regenerative medicine approaches for CKD-related cognitive decline. These represent emerging topics that may warrant monitoring in future bibliometric analyses. These innovative concepts mark a shift in research focus from merely documenting disease issues to actively seeking therapeutic approaches that can alter disease progression. Although such interventions are still in their early stages, they represent research directions not covered by traditional reviews, foreshadowing future collaborative translational research between nephrologists and neuroscientists to protect cognitive function.

Although this study systematically integrated literature from the intersection of CKD and cognitive impairment from 2005 to 2025, several limitations should be acknowledged. Despite combining searches of the WOSCC and Scopus databases and employing an automated-manual deduplication process, the study was limited to English-language literature and did not include other databases such as PubMed. As a result, non-English and gray literature may have been overlooked, potentially affecting the comprehensiveness of the conclusions. Moreover, both Scopus and WOSCC have known indexing biases toward English-language and Western-origin journals, which may lead to underrepresentation of research published in regional journals, particularly from countries such as China and Japan that are major contributors to this field. In addition, the search strategy included only articles and reviews, excluding conference abstracts, case reports, and editorials. Since review articles tend to be comprehensive and attract high citation rates, their inclusion may have introduced bias in the assessment of research influence. This study primarily relied on visualization tools including CiteSpace and VOSviewer. Differences among these tools in data preprocessing, normalization algorithms, and clustering parameters may introduce analytical biases, and no single tool can simultaneously process all types of bibliometric networks. More broadly, citation-based analyses tend to overrepresent well-established works while underestimating the influence of recent or methodologically innovative studies, and self-citation patterns may inflate the apparent impact of certain authors or research groups. Papers published in the most recent years have also had limited time to accumulate citations, which may result in underestimation of the current impact of rapidly emerging topics such as machine learning and patient-centered care. Finally, bibliometrics is a macro-level statistical method that can identify trends and patterns but cannot directly elucidate biological mechanisms or assess individual-level heterogeneity.

## 5 Conclusion

This study conducted a comprehensive bibliometric analysis of research at the intersection of CKD and cognitive impairment/dementia from 2005 to 2025. The annual number of publications increased steadily over time, with the United States and China emerging as major contributors to the field. The collaboration network showed clear geographic clustering, while thematic evolution indicated a shift from early concerns with dialysis outcomes and survival toward cognitive health, quality of life, and data-driven approaches. Machine learning, electronic health records, and patient-centered care emerged as prominent themes that may shape future research in this area.

## Data Availability

Publicly available datasets were analyzed in this study. This data can be found here: web of Science.
