# Supplementary material for: Global trends in chronic kidney disease related cognitive impairment/dementia: a bibliometric analysis (2005–2025)
Source: Front Neurol. 2026 May 19;17:1739096. doi: 10.3389/fneur.2026.1739096 (PMC13226549; doi:10.3389/fneur.2026.1739096)
Supplement: Supplementary file 1 [file Table_1.docx]

Supplementary Table S1. Full search strategies used in Web of Science Core Collection and Scopus

| **Database** | **Search field** | **Full search string** | **Filters/limits** |
| --- | --- | --- | --- |
| Web of Science Core Collection | TS (Topic) | TS = ((“Chronic Kidney Insufficien*” OR “Chronic Kidney Disease” OR “CKD” OR “Chronic Renal Disease” OR “Chronic Renal Insufficien*” OR “Kidney Failure” OR “Renal Failure” OR “Diabetic Kidney Disease” OR “Diabetic Chronic Kidney Disease” OR “Diabetic Nephropath*” OR “Diabetic Renal Disease”) AND (“cognit*” OR “cognitive impairment*” OR “cognitive decline” OR “cognitive dysfunction*” OR “cognitive deficit*” OR “neurocognit*” OR “executive function*” OR “dementia” OR “Alzheimer” OR “mild cognitive impairment” OR “MCI”)) | Language: English; Document types: Article, Review; Timespan: January 1, 2005 to March 31, 2025 |
| Scopus | TITLE-ABS-KEY | TITLE-ABS-KEY ((“Chronic Kidney Insufficien*” OR “Chronic Kidney Disease” OR “CKD” OR “Chronic Renal Disease” OR “Chronic Renal Insufficien*” OR “Kidney Failure” OR “Renal Failure” OR “Diabetic Kidney Disease” OR “Diabetic Chronic Kidney Disease” OR “Diabetic Nephropath*” OR “Diabetic Renal Disease”) AND (“cognit*” OR “cognitive impairment*” OR “cognitive decline” OR “cognitive dysfunction*” OR “cognitive deficit*” OR “neurocognit*” OR “executive function*” OR “dementia” OR “Alzheimer” OR “mild cognitive impairment” OR “MCI”)) | Language: English; Document types: Article, Review; Date range: January 1, 2005 to March 31, 2025 |
